# Case Report: Palliative chemotherapy with gemcitabine and carboplatin for carcinoma of unknown primary with metastasis in a cat

**DOI:** 10.3389/fvets.2025.1620682

**Published:** 2026-01-05

**Authors:** Hyeon-A Park, Keon Kim, Chang Hyeon Choi, Woong-Bin Ro, Chang-Min Lee

**Affiliations:** 1 College of Veterinary Medicine and BK21 FOUR Program, Chonnam National University, Gwangju, Republic of Korea; 2 Department of Veterinary Clinical Sciences, College of Veterinary Medicine, North Carolina State University, Raleigh, NC, United States

**Keywords:** carboplatin, carcinoma of unknown primary, feline, gemcitabine, supportive chemotherapy

## Abstract

Carcinoma of unknown primary (CUP) is a metastatic carcinoma in which the primary tumor site cannot be identified using standard diagnostics, including imaging, histopathology, and immunohistochemistry (IHC). In human medicine, CUP represents the sixth to eighth most prevalent form of cancer, making up about 2.3 to 5% of new cancer diagnoses. It is associated with a poor prognosis and is primarily managed with palliative chemotherapy. However, veterinary research on CUP, particularly in cats, is limited, with few reported cases. A 7-year-old spayed female Domestic Shorthair cat presented with vomiting and anorexia. Computed tomography (CT) revealed multiple masses in the abdomen, liver, and kidneys, with mild sternal and splenic hilar lymphadenopathy. Samples for histology and IHC were taken with ultrasound-guided Tru-Cut needle. The result was consistent with carcinoma of unknown primary. Given the extensive metastases and poor prognosis, a palliative approach focusing on disease stabilization and symptom management was selected. The cat was treated with gemcitabine and carboplatin, showing a positive response and maintaining quality of life for four months. Hematologic side effects, including non-regenerative anemia and neutropenia, were manageable with supportive care. This case suggests that gemcitabine-carboplatin may offer a viable palliative chemotherapy option for CUP in feline patients with non-resectable tumors. Further studies on the use of this protocol in various feline carcinomas are warranted.

## Introduction

Carcinoma of unknown primary (CUP) is a carcinoma or undifferentiated neoplasm for which a standardized diagnostic work-up fails to identify the primary tumor responsible for metastatic seeding. This standardized diagnostic work-up includes patient history, physical examination, blood analyses, computed tomography (CT) with contrast infusion, or magnetic resonance imaging (MRI), histology, and immunohistochemistry (IHC) (1). In human medicine, CUP is the seventh most frequently occurring cancer and the fourth leading cause of cancer-related deaths (2). As a result, CUP is considered highly significant, with established clinical practice guidelines for its diagnosis, treatment, and follow-up.

In human medicine, the therapeutic strategy for CUP is determined following a comprehensive evaluation of the pathological type and disease extent. Treatment goals are then personalized to the patient’s condition, aiming for local control, symptom amelioration, or prolonged survival. The approach to personalized treatment varies based on the lesion burden: patients with localized disease undergo radical treatment with curative intent, whereas those with widespread lesions receive comprehensive management centered on drug therapy (3). In human oncology, most patients diagnosed with CUP (approximately 85–90%) are classified into the unfavorable subgroup, which is associated with a poor clinical outcome and a median survival ranging from 4 to 9 months (4). Therefore, the primary realistic goals of therapy are to achieve a survival benefit and symptom palliation while preserving quality of life, rather than focusing on curing the disease. For this reason, in cases of unfavorable CUP, where options for local surgery or radiation therapy are limited or metastasis is widespread, platinum-based doublet chemotherapy is generally recommended as the standard of care according to clinical guidelines (1).

In contrast, research and publications on CUP in veterinary medicine are exceedingly rare, with only a handful of studies available. Rossi *et al*. reported a median survival of 30 days in 21 dogs with CUP, while the median survival time for dogs receiving any form of treatment was 80 days (5). Therefore, while CUP in dogs is also considered to have a poor prognosis, the current studies focus on small populations, making it difficult to determine the prevalence or effectively analyze diagnostic and treatment methods. Particularly in cats, no research on CUP has been conducted, highlighting the lack of attention this condition has received in the field. As a result, more data must be collected to provide useful diagnostic and treatment guidelines for CUP in veterinary medicine.

This case report presents palliative chemotherapy using gemcitabine and carboplatin in a feline CUP case with metastasis. To the authors’ knowledge, this is the first reported case of chemotherapy attempted in feline CUP, suggesting that gemcitabine and carboplatin could be a therapeutic option for non-resectable CUP patients.

### Case presentation

A 7-year-old spayed female Domestic Shorthair cat presented with a 10-day history of vomiting and anorexia. The cat had a history of an abdominal mass with multiple hepatic masses found in ultrasonographic evaluation at the local animal hospital.

On physical examination, the heart rate was mildly elevated at 189 beats per minute, and the respiratory rate was 56 breaths per minute. The mucous membranes appeared slightly tacky, consistent with an estimated dehydration level of 3–5%. Otherwise, the body temperature was 38.7 °C, and the systolic blood pressure was 152 mmHg, both within the normal range. Systolic pressure was measured by oscillometry and calculated as the average of the final five of seven readings, excluding the first two to minimize potential stress-related artifacts.

A complete blood count (CBC) revealed mild regenerative anemia (hematocrit 28.9%; reference range, 30.30–52.30%), with an absolute reticulocyte count of 99.2 K/uL (reference range, 3–50 K/uL) and a red blood cell (RBC) count of 6.94 M/uL (reference range, 6.54–12.2 M/uL). Serum biochemical analysis indicated a slight increase in AST (70 U/L; reference range, 0–48 U/L), while ALT, ALKP, GGT, BUN, creatinine, and phosphate remained within normal limits. Blood gas and electrolyte analysis showed hyperlactatemia (5.5 mmol/L; reference range, 0.60–2.50 mmol/L). Urinalysis showed a urine specific gravity of 1.031. Mild hematuria (1+) and trace proteinuria were detected using a dipstick. No abnormalities were identified on urine sediment examination.

CT was performed by a radiologist using a Siemens SOMATOM Emotion (16-slice CT scanner). The thorax and abdomen were scanned with the following parameters: 130 kVp, 120 mAs, and 1-mm contiguous slices. Contrast-enhanced total-body CT was performed after intravenous bolus administration of iohexol (Omnipaque 300 mg iodine/ml, 2 mL/kg). Anesthesia was induced and maintained according to a standardized protocol. For pre-medication, butorphanol (0.2 mg/kg IV) and midazolam (0.2 mg/kg IV) were administered. General anesthesia was induced with alfaxalone (2 mg/kg IV), and following induction, endotracheal intubation was performed. Anesthesia was maintained with isoflurane in oxygen.

**Figure 1:**
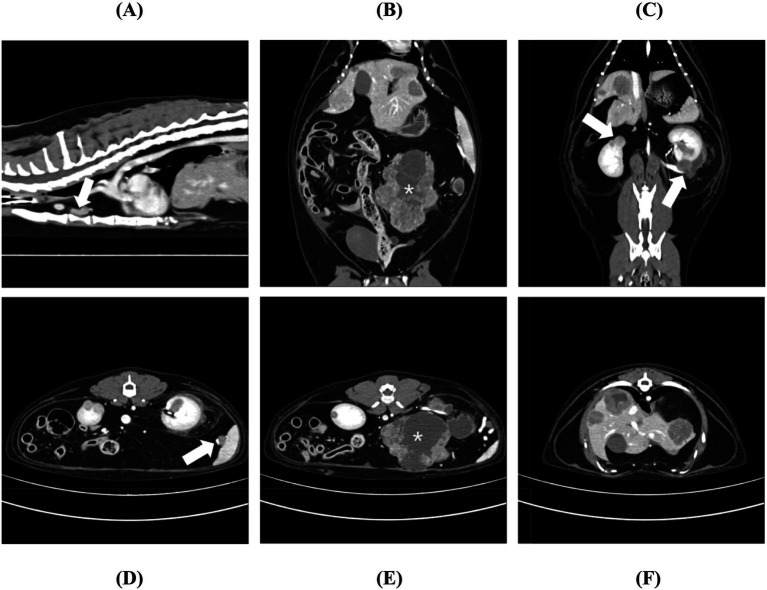
Thoracic and abdominal computed tomography, post-contrast **(A)** sagittal, **(B,C)** dorsal, and **(D–F)** transverse plan images. **(A)** Chest sagittal plane images showed mild enlargement of the sternal dorsal lymph nodes (arrow), measuring 9.2 × 5.2 mm and 11.1 × 8.5 mm. Abdominal **(B)** dorsal and **(E)** transverse plane images obtained during the portal venous phase revealed a heterogeneous mass (asterisk) measuring 75 × 100 × 45 mm, occupying a large area of the left mid-abdomen with distinctly irregular margins. No clear connection to specific parenchymal organs was identified. The margins of the mass displayed a distinct rim enhancement pattern, while the interior exhibited a heterogeneous contrast pattern with both enhancing and non-enhancing areas. **(C)** In the delayed phase, multiple hyper-attenuated masses (arrow) ranging from 2.7 mm to 25 mm were detected in both the renal medullas and outer cortical areas, showing mild and heterogeneous enhancement compared to the renal parenchyma. Notably, the lesion on the lateral aspect of the left kidney was irregularly shaped with distinctly irregular margins. Transverse plane images obtained during the portal venous phase revealed **(D)** enlargement of multiple splenic hilar lymph nodes (arrow) and **(F)** numerous heterogeneous, round masses (10–35 mm) scattered throughout the liver parenchyma. These masses exhibited mild, heterogeneous attenuation compared to the liver parenchyma and displayed distinct rim enhancement patterns.

CT revealed two distinct masses in the mid-abdomen, with the larger one measuring 100 × 75 × 45 mm. Furthermore, multiple masses were also identified in the liver and kidneys. Specifically, more than 17 heterogeneous, round masses ranging from 10 to 35 mm in diameter were detected within the liver parenchyma. In addition, at least five masses measuring 12 to 18 mm were noted on both the cranial and caudal poles of each kidney. Slight enlargement of the sternal lymph nodes and splenic hilar lymph nodes were observed (Figure 1). Ground-glass opacities were observed in the anterior lobes of both lungs. These were considered incidental findings, and the likelihood of lung metastasis was low. Additionally, remnants of the uterine body and horns with a cystic appearance were identified, which had not been observed in previous radiographic or ultrasonographic studies.

**Figure 2:**
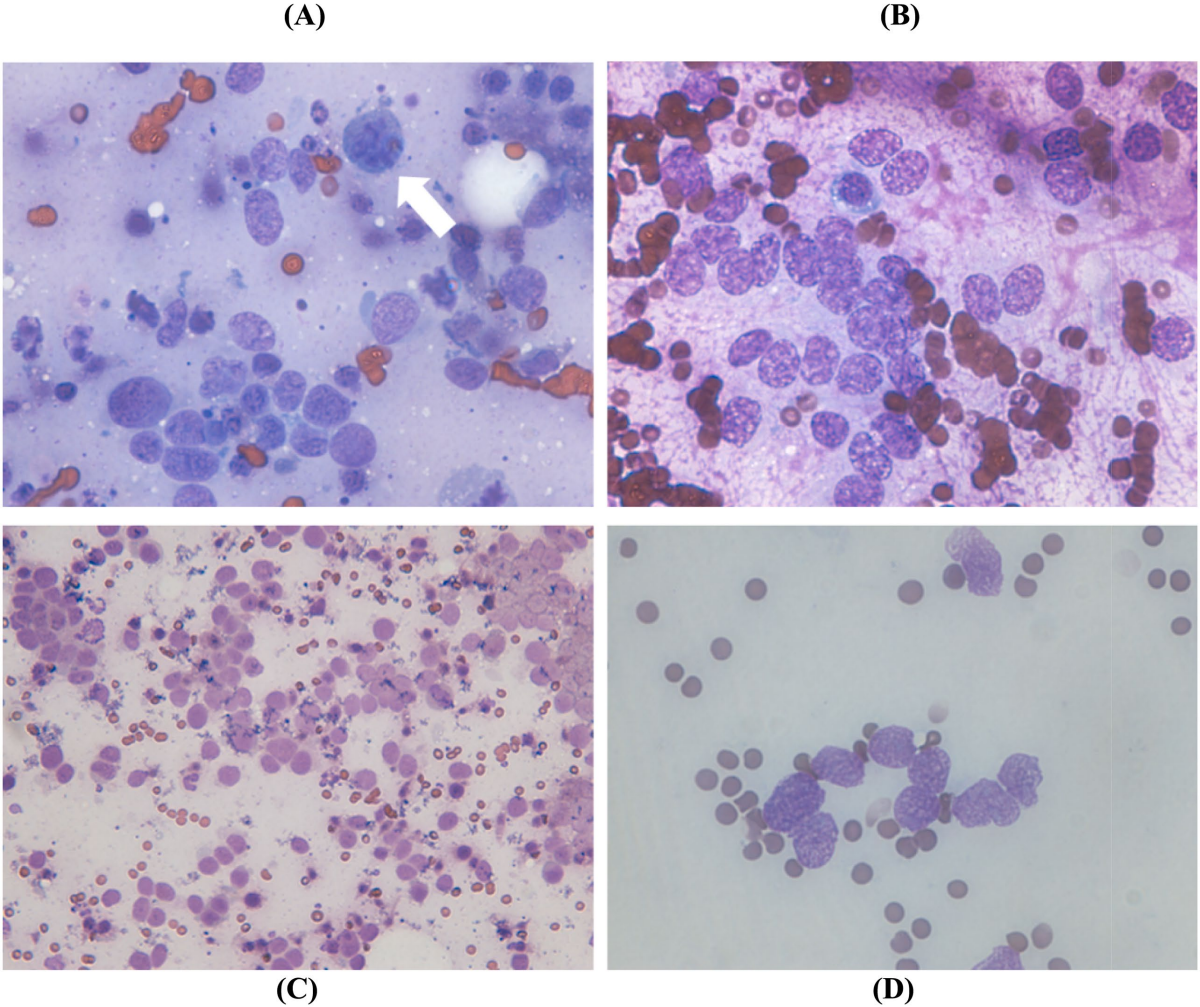
Fine needle aspiration. Abundant naked cells and epithelial cells with indistinct intercellular borders were detected in the **(A)** abdominal mass, **(B)** abdominal lymph node and **(C,D)** both kidneys. **(A)** Notable abnormalities included abnormal mitotic figure (arrow), anisokaryosis, and pleomorphism, along with scattered red blood cells and lipid droplets in the background (×1,000). Coarse nuclear features, prominent nucleoli, and nuclear vesicles were also observed in the abdominal lymph node (**B**: ×1,000) and both kidneys (**C**: ×400, **D**: ×1,000).

Fine needle aspiration (FNA) of the abdominal mass, lymph nodes, and bilateral kidneys revealed clusters of cells with indistinct intercellular borders, suggesting an epithelial origin. Cytology findings from the FNAs of the other organs had a similar appearance, confirming that the masses suspected of metastasis on CT were indeed metastatic lesions. Abnormal mitotic figures, anisokaryosis, coarse nuclei, and pleomorphism provided strong evidence of malignancy (Figure 2).

**Figure 3:**
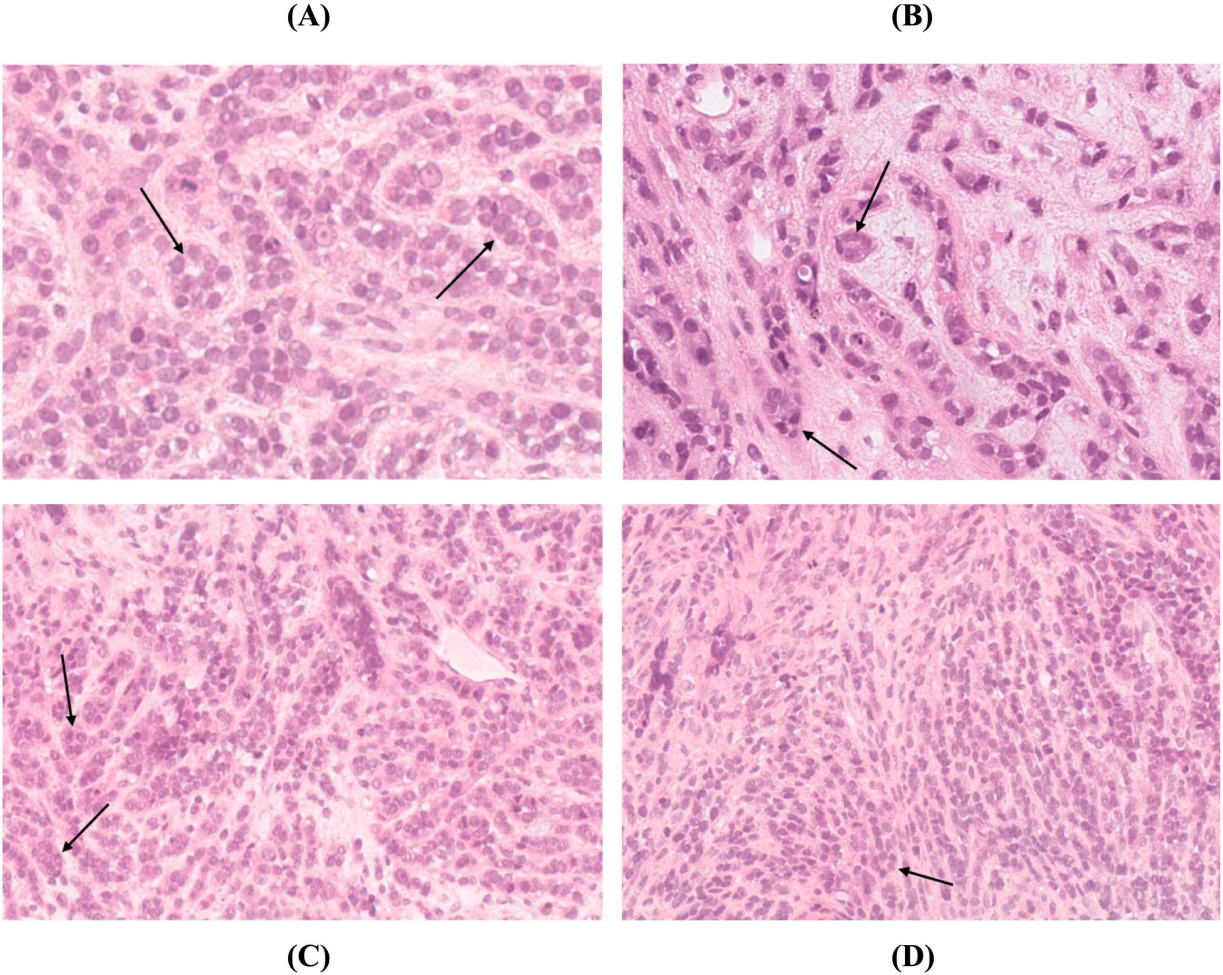
Histopathology of the **(A)** abdominal mass, **(B)** liver, **(C)** left kidney, and **(D)** right kidney. Hematoxylin and eosin stain (H&E), x40. In all tissues, neoplastic cells were similarly arranged in cords, with occasional small acinar structures (arrow). These cells were surrounded by desmoplastic connective tissue, sometimes exhibiting myxomatous characteristics. The neoplastic cells were cuboidal to ovoid to round, with scant eosinophilic cytoplasm and round to oval heterochromatic nuclei containing indistinct nucleoli.

Tissue samples were obtained from the abdominal mass, liver, and bilateral kidneys through ultrasound-guided Tru-Cut biopsy. For this procedure, a 16-gauge Tru-cut needle (Velox 2, Medax, 10 cm) was used for the liver and abdominal mass, and an 18-gauge needle of the same type and length was used for both kidneys. Histopathological examination was performed on four paraffin-embedded tissue blocks submitted from the liver, right kidney, left kidney, and abdominal mass. Sections from the liver, kidney, and abdominal mass were morphologically similar. Microscopically, these sections consisted entirely of neoplastic cells arranged in cords with occasional formation of small acinar structures, embedded in variably desmoplastic and occasionally myxomatous connective tissue. The neoplastic cells were cuboidal to ovoid to round in shape, with small amounts of eosinophilic cytoplasm and round to oval heterochromatic nuclei containing single indistinct nucleoli. Mild anisokaryosis and anisocytosis were present, and 18 mitotic figures were identified per 10 high-power fields (hpfs). Multifocal single-cell karyorrhexis and small focal areas of necrosis were also noted. No normal parenchyma was observed in the examined sections, and there was no evidence of overt vascular invasion (Figure 3).

The lesions were most consistent with a malignant epithelial neoplasm, and a diagnosis of carcinoma was made. However, despite review by three independent pathologists, the primary site of origin could not be definitively identified by consensus. Given the acinar formation and anatomical distribution, differential diagnoses included cholangiocarcinoma, exocrine pancreatic adenocarcinoma, renal carcinoma, and malignant granulosa cell tumor. A neuroendocrine carcinoma was considered less likely. Immunohistochemical staining was recommended to further classify the neoplasm. The pathologist advised the use of CK19, CK7, chromogranin A, Tamm-Horsfall protein and glutathione S-transferase on the abdominal mass sample. However, due to the poorly differentiated nature of the neoplasm, the pathologist noted that IHC might not definitively determine the tumor’s origin.

Immunohistochemistry (IHC) revealed positive staining for Tamm-Horsfall protein (THP) and glutathione S-transferase-*α* (GST-α), while cytokeratin 19, cytokeratin 7, and chromogranin A were negative. These negative markers reduced the likelihood of cholangiocarcinoma, transitional cell carcinoma, and neuroendocrine tumor. Serum estrogen (E2) concentrations were within the normal range for spayed female cats (<5.0 pg/mL; reference <15 pg/mL), making a granulosa cell tumor unlikely. However, the overall IHC profile was non-specific, and the very low degree of tumor differentiation limited the ability to determine the tissue of origin. Based on the integration of imaging, histopathology, and IHC findings, the case was diagnosed as carcinoma of unknown primary (CUP) with multiple-organ metastasis.

In this case, because the primary site could not be determined among the various possible carcinoma types, the key focus was to find an effective chemotherapy regimen for the multiple tumors. Given the patient’s extensive metastases, we prioritized palliative chemotherapy to preserve quality of life, minimize toxicity, and palliate symptoms, anticipating only a modest survival benefit. The carboplatin and gemcitabine protocol was administered based on evidence from human medicine of its established efficacy against various solid tumors and its therapeutic activity in CUP. Furthermore, it was selected for its favorable tolerability profile. Although nausea and vomiting were the most common adverse events, they were generally mild in severity, and the rate of Grade 3–4 hematologic toxicity was less than 15% (6). The combination protocol of carboplatin and gemcitabine consisted of a 21-day repeating cycle. Within a single cycle, 2 mg/kg gemcitabine (Gemja; Boryung Pharmaceuticals, Seoul, Korea) was administered via a 20-min intravenous infusion on days 1 and 8. In addition, 10 mg/kg carboplatin (Neoplatin; Boryung Pharmaceuticals, Seoul, Korea) was given intravenously on day 1, 4 h after the administration of gemcitabine. Day 15 was designated as a scheduled treatment break, during which a CBC was performed for monitoring.

With the exception of a few adverse effects, the patient tolerated well the chemotherapy for 4 months, despite multiple metastases following the initiation of this chemotherapy protocol. On Day 55, the patient developed Grade 4 neutropenia (0.12 K/μL, reference range, 2.3–10.29 K/μL) accompanied by Grade 1 mild fever (39.7 °C), according to Veterinary Comparative Oncology Group-Common Terminology Criteria for Adverse Events v2 (VCOG-CTCAE v2) (7). Recombinant filgrastim (Leucostim; Dong-A ST Co., Ltd., Seoul, Korea) was administered subcutaneously at a dose of 5 μg/kg. Broad-spectrum antibiotics were concurrently initiated, including cefazolin (Cefazoline Inj.; Chong Kun Dang Pharmaceutical Corp., Seoul, Korea, 20 mg/kg IV) and enrofloxacin (Baytril Inj.; Bayer Korea, Seoul, Korea, 5 mg/kg IM) administered in-hospital, followed by oral amoxicillin-clavulanate (Amocla Tab.; Kukje Pharma, Seongnam, Korea, 12.5 mg/kg PO BID for 7 days) as discharge medication. On day 8 after initiating this treatment, follow-up bloodwork revealed a neutrophil count of 9.64 K/μL (reference range, 2.3–10.29 K/μL), and body temperature had normalized to 39.0 °C. CBC monitoring was continued at each subsequent visit, and no additional episodes of neutropenia were observed.

On Day 63, the patient subsequently developed Grade 3 anemia (Hct 17.9%; reference range, 30.3–52.3%). The cat exhibited mildly decreased activity and clinical signs including tachycardia (heart rate: 200 beats/min) and hypotension (mean systolic blood pressure: 92 mmHg) on physical examination. Blood typing confirmed type A and, following a negative cross-match test, a whole blood transfusion was performed using a unit of whole blood sourced from a feline blood bank. In addition, BUN and creatinine levels remained within normal limits until Day 55. Subsequently, the patient developed mild azotemia consistent with International Renal Interest Society (IRIS) AKI Grade 2. The azotemia progressed to IRIS AKI Grade 3 by Day 91 and became severe during the terminal stage of the disease (Day 112), with a BUN of 83 mg/dL (reference range, 16–36 mg/dL) and creatinine of 5.0 mg/dL (reference range, 0.8–2.4 mg/dL). According to VCOG-CTCAE v2, this value corresponds to a Grade 3 adverse event.

**Figure 4:**
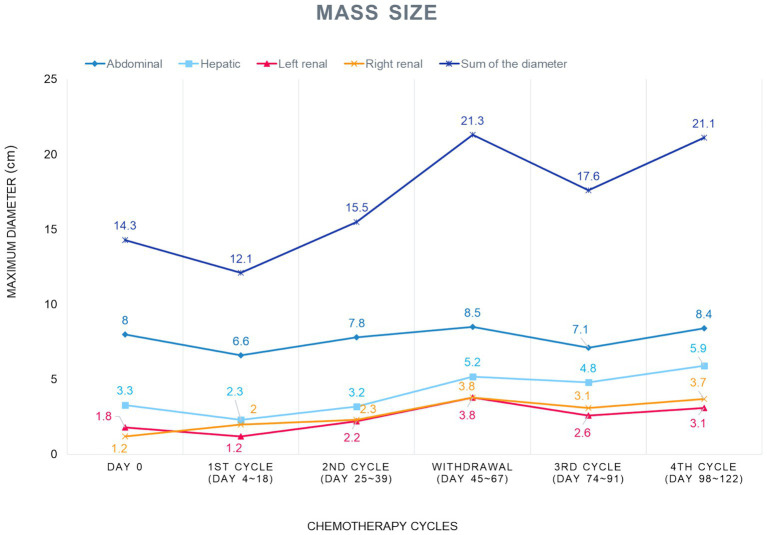
Changes in the sum of the longest diameters (SLD) of target lesions during the gemcitabine-carboplatin combination protocol. Abdominal ultrasonography was used for serial monitoring. For response assessment, four target lesions were selected by choosing the largest measurable lesion in each organ: abdominal mass #1, hepatic mass #1, left renal mass #1, and right renal mass #1. The overall tumor burden, represented by the SLD, is plotted for each cycle. A decreasing trend in the SLD was shown in the 1st and 3rd cycles, whereas an increasing trend was noted in the 2nd cycle and during treatment delay.

Tumor response to chemotherapy was evaluated based on the principles of the Response Evaluation Criteria for Solid Tumours in Dogs v1.0 (cRECIST v1.0) (8). Although CT or MRI are the standard imaging modalities for cRECIST v1.0, abdominal ultrasonography was utilized in this case for serial monitoring. Therapeutic efficacy was assessed by tracking changes in the sum of the longest diameters (SLD) of designated target lesions. Specifically, four target lesions were defined by selecting the largest measurable mass in each organ: the abdominal mass (abdominal mass #1), a representative hepatic lesion (hepatic mass #1), and the largest cortical masses in each kidney (left renal mass #1 and right renal mass #1). These four lesions served as the basis for calculating the SLD and monitoring tumor progression over time (Figure 4).

The baseline SLD was 14.3 cm. After the initial administration of the gemcitabine and carboplatin combination, the SLD decreased to 12.1 cm, a reduction of approximately 15%, suggesting a positive response to chemotherapy. However, after the first chemotherapy cycle, the total diameter increased from 12.1 cm to 15.5 cm, a size increase of approximately 28%. Subsequently, during a three-week delay of treatment after the second cycle due to neutropenia, anemia and the owner’s personal reasons, the tumor size further increased from 15.5 cm to 21.3 cm, a 37% rise.

Nevertheless, immediately after the third cycle began, the total diameter rapidly decreased from 21.3 cm to 17.6 cm, a reduction of approximately 17%, indicating another good response to the chemotherapy. Considering the entire treatment course, the chemo-responsiveness was generally positive, excluding the periods of treatment delay. However, during another two-week treatment delay in the fourth cycle due to azotemia, the tumor size increased again from 17.6 cm to 21.1 cm. Subsequently, the patient passed away naturally at home on Day 123 (Table 1).

**Table 1:** Timeline of chemotherapy, serial ultrasonographic measurements of target lesions, and clinical events.

Parameter	Day 0	Day 4	Day 11	Day 18	Day 25	Day 32
Abdominal mass (cm × cm)	8×4.5		6.6×3.9			7.8×5.1
Hepatic mass (cm × cm)	3.3×2.7		2.3×2.1			3.2×3.1
Left renal mass #1 (cm × cm)	1.8×1.6		1.2×1.1			2.2×2.1
Right renal mass #1 (cm × cm)	1.2×1.0		2.0×1.65			2.3×1.0
Sum of diameters (cm)	14.3		12.1			15.5
Clinical course						
Tx.		Gemcitabine + carboplatin	Gemcitabine	Scheduled treatment break	Gemcitabine + carboplatin	Gemcitabine

	Day 39	Day 46	Day 55	Day 63	Day 74	Day 84	Day 91
Abdominal mass (cm × cm)					8.5×4.9	7.1×3.6	
Hepatic mass (cm × cm)					5.2×4.8	4.8×3.5	
Renal mass (L) (cm × cm)					3.8×3.1	2.6×2.6	
Renal mass (R) (cm × cm)					3.8×3.5	3.1×2.8	
Sum of diameters (cm)					21.3	17.6	
Clinical course			Neutropenia (NEU 0.12 K/uL), fever	Non-regenerative anemia (Hct 17.9%)			
Tx.	Scheduled treatment break	Treatment delay due to the owner’s personal reason	Treatment delay, Antibiotics, G-CSF	Treatment delay, Transfusion (Hct 17.9 ➔ 21.8)	Gemcitabine + carboplatin	Gemcitabine	Scheduled treatment break

	Day 98	Day 105	Day 112	Day 115	Day 121	Day 123
Abdominal mass (cm × cm)			8.4			
Hepatic mass (cm × cm)			5.9×4.1			
Renal mass (L) (cm × cm)			3.1×2.7			
Renal mass (R) (cm × cm)			3.7×2.9			
Sum of diameters (cm)			21.1			
Clinical course		Azotemia (BUN 66 mg/dL, CREA 3.3 mg/dL)	Azotemia (BUN 83 mg/dL, CREA 5.0 mg/dL)			Patient passed away
Tx.	Gemcitabine + carboplatin	Treatment delay, Fluid therapy	Treatment delay, Fluid therapy	Gemcitabine	Scheduled treatment break	

Dimensions for each target lesion are reported as long × short axis (cm) on abdominal ultrasonography, while the sum of longest diameters (SLD, cm) represents the sum of the longest diameters in accordance with VCOG canine RECIST v1.0 criteria. The four target lesions included an abdominal mass (abdominal mass #1), a representative hepatic parenchymal lesion (hepatic lesion #1), and cortical masses in the left and right kidneys (left renal lesion #1 and right renal lesion #1). Day 0 denotes the baseline (pre-treatment) assessment. The scheduled treatment break represents a protocol-planned rest period during which a CBC was performed for monitoring. Treatment delay refers to unscheduled interruptions caused by adverse events or owner-related constraints (e.g., febrile neutropenia on Day 55; non-regenerative anemia on Day 63 requiring transfusion, Hct 17.9% → 21.8%; azotemia on Days 105 and 112 managed with fluid therapy). The SLD decreased after chemotherapy (14.3 cm at baseline → 12.1 cm on Day 11), increased during treatment delay (reaching a peak of 21.3 cm on Day 74), and decreased again after treatment re-initiation (17.6 cm on Day 84). The patient died naturally at home on Day 123. Abbreviations: NEU, absolute neutrophil count; Hct, hematocrit; L/R, left/right; Tx, treatment.

At the start of chemotherapy, the owner’s priority was to keep the cat comfortable at home and avoid prolonged hospitalization. After being informed that a cure was unlikely, the owner consented to palliative chemotherapy, aiming for symptom relief and additional time with acceptable side effects. During treatment, the owner observed improved appetite and energy and was generally satisfied with the course of therapy, feeling that the overall plan prioritized the cat’s quality of life. Although we proposed a necropsy to determine the primary origin of the tumor, we agreed with the owner not to proceed after they declined, despite the persistent uncertainty.

## Discussion

Determining the primary site of metastatic tumors is a significant challenge in clinical oncology. Annually in the United States, an estimated 100,000 malignancies are of uncertain origin. These neoplasms may appear entirely undifferentiated or may be relatively well-differentiated, yet they present without a discernible primary source. Although the standard diagnostic approach relies on clinical data, radiological findings, and histopathology, a significant number of cases remain unclassified even after exhaustive investigations involving blood tests, advanced imaging, endoscopy, and molecular evaluation. Such cases are designated as cancer of unknown primary, which constitutes an estimated 31,000 new diagnoses annually in the United States (9).

In human oncology, pathology and IHC remain the gold standard for the diagnostic workup aimed at identifying the primary site of a metastatic tumor. When the primary site cannot be determined even after this process, the case is classified as CUP. IHC is a key tool for defining tumor type, subtype, site of origin by assessing specific protein expression in tissue samples. However, even when combined with light microscopy, the tissue of origin can be identified in only ~30% of CUP cases (3). Although the development of new antibodies has expanded the pathologist’s armamentarium and recent efforts to systematize IHC staining have reduced subjectivity, IHC still lacks uniform specificity and sensitivity. Consequently, a subset of metastatic cancers with occult primaries remains unresolved despite exhaustive IHC workups (9).

Similarly, in this patient, the primary origin of the tumor could not be identified despite a comprehensive evaluation that included patient history, physical examination, blood analyses, contrast-enhanced CT, histopathology, and immunohistochemistry. At the initial presentation, the symptoms and physical examination findings were non-specific. Serum chemistry tests revealed no remarkable abnormalities other than a mild elevation of AST. Urinalysis also showed no specific findings apart from mild hematuria. A CT scan was performed to locate the primary site; however, the tumor had already disseminated to the abdominal cavity, liver, and kidneys at the time of the scan. The largest mass, located in the abdominal cavity, did not show any connection to any organ, making it impossible to presume the primary site from the CT findings.

Furthermore, the FNA and biopsy results indicated an epithelial origin, but the primary site could not be inferred because the tumor was extremely poorly differentiated. Immunohistochemistry (IHC) provided some information to narrow the differential diagnoses; however, the overall staining pattern was non-specific and insufficient to determine the tissue of origin. Based on the integrated assessment of imaging, histopathology, and IHC findings, a definitive primary site could not be established, and the case was therefore diagnosed as carcinoma of unknown primary (CUP) with multiple-organ metastasis. In this patient, carcinoma was considered the most likely tumor category, and due to the extensive metastatic burden, surgical intervention was deemed not feasible, leading to the selection of palliative chemotherapy. Because the list of potential differentials—including renal carcinoma, cholangiocarcinoma, mesenteric carcinoma, and ovarian carcinoma—was broad, it was important to choose a chemotherapeutic protocol with activity across multiple carcinoma types, consistent with CUP treatment principles. The therapeutic goals were to maintain quality of life, manage clinical signs, and prolong survival within an acceptable toxicity profile.

The gemcitabine-carboplatin combination has demonstrated synergistic effects, with proven clinical efficacy and tolerability across various carcinomas in humans, including cholangiocarcinoma, ovarian, pancreatic, lung, and thyroid gland cancers (10). It has also been reported to have very good tolerability and significant efficacy in patients with carcinoma of unknown primary (6), and therefore was considered a reasonable treatment option in the present case, which had a broad range of differential diagnoses. In terms of overall safety, a study in cats with naturally occurring carcinoma reported acceptable toxicity profiles with this combination, with adverse events including Grade 1 gastrointestinal signs in 50% of cases and neutropenia in 28% (Grade 2, 14%; Grade 3, 5%; Grade 4, 7%) (11). In addition, it has been reported to show low toxicity even in patients with impaired renal function and to have no dose restrictions according to renal function (12), so it was considered a relatively safe protocol even for the present patient, whose kidneys were significantly burdened by tumor invasion.

This is consistent with the differences in nephrotoxicity profiles among platinum agents. Cisplatin is known to cause dose-limiting nephrotoxicity mediated by proximal tubular necrosis and oxidative injury, and it is contraindicated in cats due to severe systemic and pulmonary toxicity. In contrast, carboplatin is a second-generation analogue developed to reduce the major toxicities of cisplatin, and it is regarded as a drug with markedly lower nephrotoxicity (13). In a phase I clinical trial in cats, the dose-limiting toxicity of carboplatin was identified as myelosuppression, and no clinically meaningful nephrotoxicity was reported, suggesting that carboplatin has a relatively safe renal toxicity profile in cats (13).

CUP is generally considered to have a poor prognosis in both human and canine patients. In dogs specifically, the reported MST for CUP is short, ranging from approximately 30 days (5). In light of this, the patient in this case survived for 123 days, a noteworthy outcome. This survival time, which exceeds typical expectations, suggests that the chemotherapy protocol was beneficial in mitigating disease progression and inhibiting further metastasis, despite its limitations. The most important evidence for this positive evaluation is the clear tumor responsiveness confirmed by objective data during the treatment course. At the start of treatment (Day 0), the sum of the tumor diameters was 14.3 cm, which decreased to 12.1 cm after the first chemotherapy cycle, demonstrating a clear initial response. More notably, after the tumor size sharply increased to a peak of 21.3 cm during a period of treatment delay, it again decreased to 17.6 cm upon resumption of chemotherapy in the third cycle. This distinct cyclical pattern—shrinkage during treatment and regrowth during periods of treatment delay—serves as strong evidence that the tumor possessed a clear sensitivity to the gemcitabine-carboplatin protocol, even though the disease ultimately progressed.

However, two major limitations complicated the final evaluation and interpretation of this responsiveness. First, frequent and prolonged periods of treatment delay, necessitated by hematologic side effects and the owner’s circumstances, led to tumor regrowth, resulting in an overall classification of progressive disease (PD) according to cRECIST v1.0 guidelines (8). Second, due to the risks associated with repeated anesthesia, follow-up imaging was performed with abdominal ultrasonography rather than CT, which constrained the quantitative and precise assessment of treatment response. Because ultrasonographic measurements are known to be subject to interobserver variability, we sought to minimize this limitation by performing all examinations with the same machine and the same experienced operator at all time points.

Furthermore, during the course of chemotherapy, the patient developed Grade 4 neutropenia (0.12 K/μL) and a Grade 1 mild fever (39.7 °C), which was classified as febrile neutropenia according to VCOG-CTCAE v2 (7). In accordance with febrile neutropenia management guidelines, broad-spectrum antibiotics such as cefazolin and enrofloxacin were administered during hospitalization, followed by a 7-day course of oral amoxicillin-clavulanate (14).

Additionally, a single subcutaneous dose of recombinant filgrastim (rhG-CSF) was administered. In general, the standard approach for febrile neutropenia consists of broad-spectrum antibiotics and close monitoring, and rhG-CSF is not routinely indicated (15). However, in this patient, several factors warranted consideration of rhG-CSF. The patient had already experienced a treatment delay in the preceding week due to owner-related circumstances. Given the importance of maintaining the chemotherapy schedule in a patient with widespread metastatic disease, further interruption was considered potentially disadvantageous. Therefore, rhG-CSF was administered to accelerate neutrophil recovery and minimize additional treatment delays. In addition, the owner wished to minimize hospitalization, making rapid hematologic stabilization and recovery of neutrophil counts particularly important. Based on previous feline studies reporting that rhG-CSF was effective in neutropenia caused by viral infections or drug exposure (16–18), a single supportive dose was considered an effective means of promoting marrow recovery.

However, the use of rhG-CSF for chemotherapy-induced neutropenia in cats remains off-label and is not regarded as standard therapy (19). In particular, prolonged administration (≥2 weeks) may induce neutralizing antibodies and paradoxically lead to persistent neutropenia (17). To minimize this risk, a single-dose strategy was selected instead of repeated administration. Thereafter, hematologic changes were monitored at each hospital visit through CBC testing. By Day 8, the neutrophil count had recovered to 9.64 K/μL and the body temperature had normalized to 39.0 °C, demonstrating the effectiveness of this therapeutic approach. No evidence of rebound neutropenia or persistent neutropenia was observed after rhG-CSF administration, indicating that antibody-mediated adverse effects did not occur.

On Day 63, the patient developed Grade 3 acute anemia (Hct 17.9%) accompanied by clinical signs of acute lethargy, tachycardia, and hypotension. In cats, routine transfusion is generally not recommended due to risks such as transfusion reactions (e.g., febrile non-hemolytic transfusion reactions, allergic reactions) and alloantibody formation. Therefore, transfusion is reserved for life-threatening anemia after a careful risk–benefit assessment and appropriate blood typing and cross-matching (20). Although standardized transfusion guidelines are lacking in feline oncology, a transfusion was indicated for this patient because the benefits were judged to outweigh the risks of adverse effects, given the life-threatening acute clinical signs. Accordingly, blood typing and cross-matching were performed prior to the transfusion, which was well tolerated without notable adverse effects.

Additionally, because this patient had substantial renal lesion burden in both kidneys, the interpretation of azotemia required consideration of both tumor-related renal involvement and potential chemotherapy-associated nephrotoxicity. In this context, the temporal pattern of azotemia progression corresponded more closely with the gradual enlargement of bilateral renal lesions than with the timing of carboplatin administration. Serum urea and creatinine concentrations remained within their respective reference intervals through Day 55, despite multiple chemotherapy cycles, but azotemia was first detected only after ultrasonography confirmed increased renal mass size. The azotemia subsequently progressed to IRIS AKI Grade 3 by Day 91 in the later stage of disease. These findings support the likelihood that functional renal impairment due to extensive renal involvement contributed more substantially to the development of azotemia, and suggest that the overall nephrotoxic risk of this protocol was comparatively low. Even so, the potential contribution of carboplatin-associated nephrotoxicity cannot be completely excluded and remains a limitation of this report.

In summary, the gemcitabine-carboplatin protocol can be evaluated as a successful palliative therapeutic strategy in this case in terms of both efficacy and safety. First, it demonstrated objective therapeutic efficacy. A clear sensitivity to the drugs was confirmed, with a distinct decrease in the total tumor diameter during the first and third treatment cycles. Second, the protocol showed excellent safety and tolerability without severe, unmanageable side effects. Despite multiple metastases, the patient maintained a good quality of life for four months without vomiting or anorexia. Clinically, no significant adverse effects were observed other than a mild Grade 1 fever. Although hematologic toxicity occurred, it was at a level that could be effectively managed with supportive care. Moreover, despite the burden of bilateral renal tumors, azotemia was stably controlled except in the terminal stage, indicating low concern for nephrotoxicity.

Therefore, this case suggests that the gemcitabine-carboplatin protocol can be an effective palliative chemotherapy option for patients with metastatic CUP, capable of preserving quality of life. Further studies with a larger cohort are warranted to maximize the therapeutic potential of this regimen and to establish optimal dosing and administration schedules for various feline carcinomas.

## Data Availability

The original contributions presented in the study are included in the article/supplementary material, further inquiries can be directed to the corresponding author/s.
